# Study of Disease Progression and Relevant Risk Factors in Diabetic Foot Patients Using a Multistate Continuous-Time Markov Chain Model

**DOI:** 10.1371/journal.pone.0147533

**Published:** 2016-01-27

**Authors:** Alexander Begun, Stephan Morbach, Gerhard Rümenapf, Andrea Icks

**Affiliations:** 1 Paul Langerhans Group for Health Services Research and Health Economics, German Diabetes Center, Heinrich-Heine University, Düsseldorf, Germany; 2 Department of Diabetes and Angiology, Marienkrankenhaus, Soest, Germany; 3 Department of Vascular Surgery, Gefässzentrum Oberrhein, Diakonissen-Stiftungs-Krankenhaus Speyer, Mannheim, Germany; 4 Department of Public Health, Center of Health and Society, Heinrich-Heine University, Düsseldorf, Germany; University of Louisville, UNITED STATES

## Abstract

The diabetic foot is a lifelong disease. The longer patients with diabetes and foot ulcers are observed, the higher the likelihood that they will develop comorbidities that adversely influence ulcer recurrence, amputation and survival (for example peripheral arterial disease, renal failure or ischaemic heart disease). The purpose of our study was to quantify person and limb-related disease progression and the time-dependent influence of any associated factors (present at baseline or appearing during observation) based on which effective prevention and/or treatment strategies could be developed. Using a nine-state continuous-time Markov chain model with time-dependent risk factors, all living patients were divided into eight groups based on their ulceration (previous or current) and previous amputation (none, minor or major) status. State nine is an absorbing state (death). If all transitions are fully observable, this model can be decomposed into eight submodels, which can be analyzed using the methods of survival analysis for competing risks. The dependencies of the risk factors (covariates) were included in the submodels using Cox-like regression. The transition intensities and relative risks for covariates were calculated from long-term data of patients with diabetic foot ulcers collected in a single specialized center in North-Rhine Westphalia (Germany). The detected estimates were in line with previously published, but scarce, data. Together with the interesting new results obtained, this indicates that the proposed model may be useful for studying disease progression in larger samples of patients with diabetic foot ulcers.

## Introduction

Macro- and microvascular complications (peripheral arterial disease (PAD), ischaemic heart disease (IHD) and chronic renal failure (CRF)) increase both the morbidity and mortality of diabetic patients. One of the most complex and economically challenging complications of these patients is foot ulceration. About 15% of all patients with diabetes experience at least one episode of this complication during their lifespan [1]. Without provision of preventative measures such as adapted shoes and insoles, and regular podiatric care, following the initial “healing”, a diabetic patient with a history of a foot lesion will have more than one relapse per year [2–3]. Thus, diabetic foot syndrome should be considered a lifelong disease with patients being “in remission” rather than “cured” after the initial healing of an index diabetic foot wound [4]. More than 80% of all diabetes-related amputations are preceded by an ulcer [5]. Hence every new episode potentially increases the risk of lower extremity amputation [6], increases the associated costs [7] and the risk of premature death [8,9]. Recent publications suggest that the prognosis of patients with diabetic foot ulcers is similar to many cancers [10,11]. Amputation- or ulcer-free survival may therefore reflect the effectiveness of diabetic foot ulcer management strategies more effectively [12].

However, there are few quantitative data available about the dynamics of disease progression and the effects on this of demographic variables such as age or gender, or of additional risk conditions, prevalent either at baseline or appearing during the course of the disease. (e.g. PAD, IHD, CRF; definitions: see Table 1). Reliable knowledge about the effect of those risk factors could result in more effective strategies for the prevention and management of patients with diabetic foot ulcers.

**Table 1:** Distribution of the patients at first presentation by gender, age, previous ulcer and amputation, and comorbidities.

Gender	Number (%)	Min age (y)	Mean age (SD)	Ulcer before (%)	Minor amputation before (%)	Major amputation before (%)	PAD before (%)	CRF before (%)	IHD before (%)
Men	154 (59.2)	25.5	66.9 (10.0)	89 (57.8)	38 (24.7)	9 (5.8)	62 (57.8)	43 (27.9)	32 (20.8)
Women	106 (40.8)	27.2	72.0 (11.3)	57 (53.8)	17 (16.0)	4 (3.8)	89 (58.5)	18 (17.0)	20 (18.9)
Total	260 (100.0)	25.5	69.0 (10.9)	146 (56.2)	55 (21.2)	13 (5.0)	151 (58.1)	61 (23.5)	52 (20.0)

**Definitions:** Peripheral arterial disease (PAD): ankle-brachialpressure index (ABI),0.9 with additional investigation by means of duplex ultrasonography or angiography.; Chronic Renal Failure (CRF): serum creatinine concentration ≥1.5mg/dL; Ischaemic Heart Disease (IHD): history of angina pectoris or myocardial infarction, any positive cardiac stress test result, or pathological signs on coronary angiography.

Multistate Markov chain models assume that the time course of chronic diseases can be described by assigning every individual patient to a distinct state at any defined time and that the next state depends on the current but not the preceding states. So transition characteristics and the influence of risk factors (predictors) can be estimated using the maximum likelihood or the Bayesian estimator.

In the present study we therefore developed a nine-state continuous-time Markov chain model for quantifying disease progression and the time-dependent influence of relevant risk factors for diabetic foot patients and then tested this model on real world data. Results obtained in this paper allow us to better understand complex interplay of active and inactive episodes of diabetic foot disease.

## Materials and Methods

### The study data

**Fig 1:**
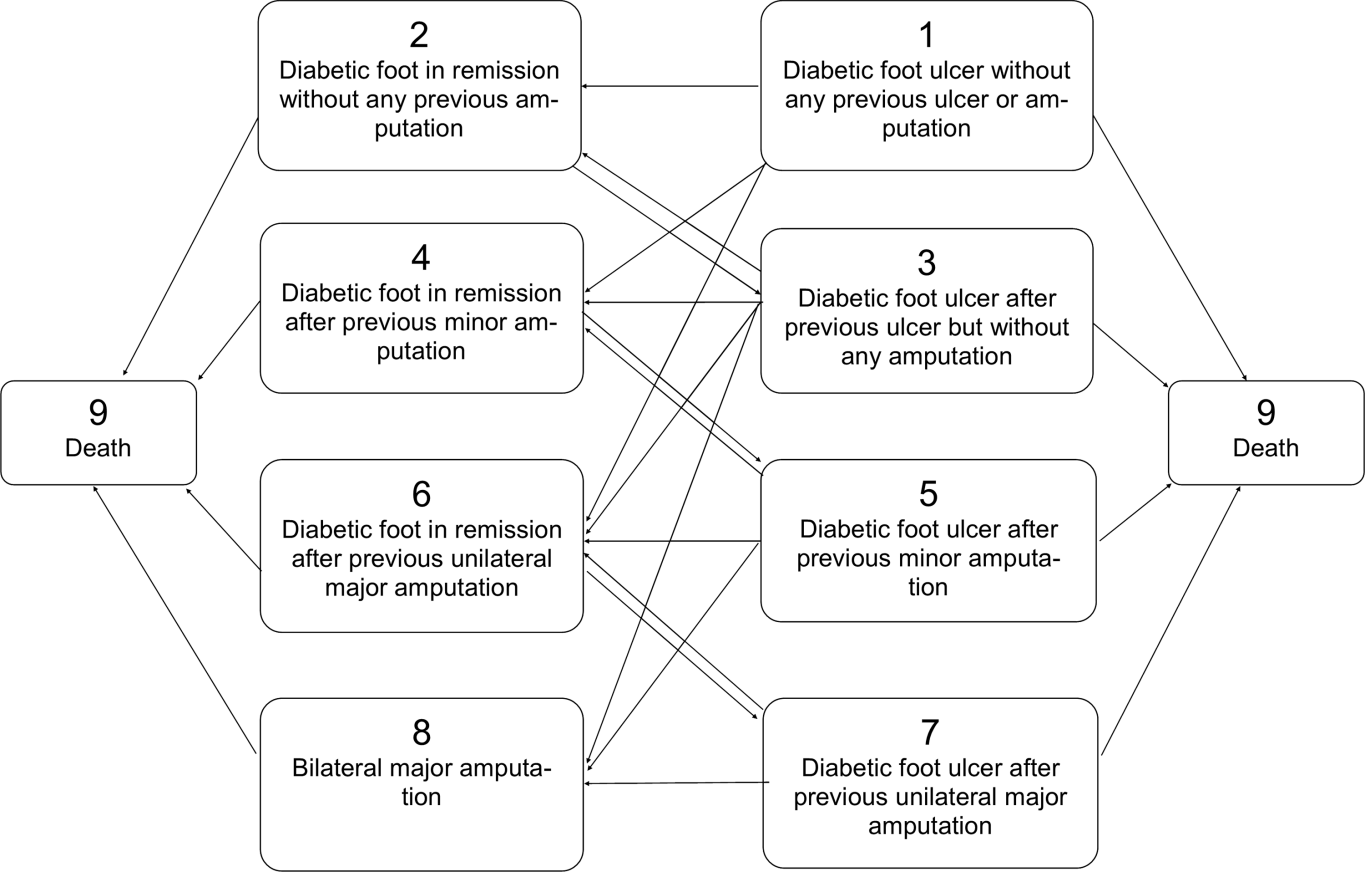
A nine-state Markov model for diabetic foot disease.

The long-term data of 260 consecutive patients presented between June 1998 and December 1999 with a new diabetic foot ulcer to a single specialized diabetic foot center in North-Rhine Westphalia (Germany) were used for analysis. Patients were followed either until December 31, 2014 or death. The date of the last contact with each patient (right censoring) was documented. For all patients, data which were relevant to the course of the diabetic foot disease (outcomes as well as predictors) were documented in the treating center with exact dates. All patients gave informed consent for participation in the study. They agreed to be contacted personally or allowed the investigators to obtain information on their outcomes from their relatives or their family physicians. A part of this cohort (247 patients without previous unilateral major amputation at study inclusion) has been described in detail elsewhere [13]. All living patients were divided into eight groups on the basis of their ulcer history (previous or current) and amputation status (none, minor or major), adding death as an absorbing ninth state (see Fig 1).

Patients in states 1, 3, 5, and 7 had by definition an active ulcer, while those in stages 2, 4, 6, and 8 were considered “in remission” [4]. Patients could switch between the groups several times during the follow-up period. Potential risk factors used for the parameter estimation included time-independent (gender) and time-dependent (age, PAD, CRF, and IHD) factors.

### Model description

On the supposition of full observability, the nine-state model can be decomposed into eight competing risks survival submodels (the absorbing state death cannot be an origin state). An additional assumption of the proportional hazards allows us to estimate the influence of risk factors on transition intensities using Cox-like regression. All unknown parameters were estimated using the maximum likelihood method.

Since all transitions are fully observable (the dates of departure from the origin state and arrival into the destination state or censoring were known), we separately analyzed the groups of transitions having common origin states. For any origin state *i* we defined the set of possible destination states by a subset *D*_*i*_ ⊂ *D*_0_ = {1,2,3,4,5,6,7,8,9}. Under the settings of the competing risks model with proportional hazards [14], [15] the intensity of transition (hazard function) from state *i* to state *j*, *j* ∈ *D*_*i*_, at age *t* was defined as

$$\lambda_{i,j}(t;\beta_{i,j},u_{t})=\text{exp}(\beta_{i,j}{}^{T}u_{t})\lambda_{0,i,j}(t),$$ (1)

where *β*_*i*,*j*_^*T*^ is transposed vector of the Cox-regression coefficients, *u*_*t*_ is the vector of time-dependent explanatory factors (covariates), and *λ*_0,*i*,*j*_(*t*) stands for the baseline hazard functions. If the destination state is a unique element of *D*_*i*_ then the full hazard function can be calculated as follows

$$\lambda_{i}(t;\beta_{i,1},\ldots ,\beta_{i,|D_{i}|},u_{t})=\sum_{j\in D_{i}}\text{exp}(\beta_{i,j}{}^{T}u_{t})\lambda_{0,i,j}(t).$$ (2)

Here |*D*_*i*_| denotes the number of possible transitions from state *i*. Note that

$$\nu_{i,j}(t;\beta_{i,1},\ldots ,\beta_{i,|D_{i}|},u_{t})=\frac{\lambda_{i,j}(t;\beta_{i,j},u_{t})}{\lambda_{i}(t;\beta_{i,1},\ldots ,\beta_{i,|D_{i}|},u_{t})}$$ (3)

is the conditional probability of the transition from state *i* in destination state *j* at time *t*.

For an individual that arrives in the origin state *i* at time *t*_0,*i*_ we can calculate the conditional (left truncated) survival function using the formula

$$S_{i}(t;\beta_{i,1},\ldots ,\beta_{i,|D_{i}|},u_{t}\;|\;t_{0,i})=\text{exp}\left(-\int_{t_{0,i}}^{t}\lambda_{i}(\tau {;}_{i,1},\ldots ,\beta_{i,|D_{i}|},u_{\tau })d\tau \right).$$ (4)

The conditional probability density function corresponding to the transition from state *i* to state *j* at time *t* is equal to

$$f_{i,j}\;(t;\beta_{i,1},\ldots ,\beta_{i,|D_{i}|},u_{t}\;|\;t_{0,i})=\lambda_{i,j}\;(t;\beta_{i,j},u_{t})\text{exp}\left(-\int_{t_{0,i}}^{t}\lambda_{i}\;(\tau {;}_{i,1},\ldots ,\beta_{i,|D_{i}|},u_{\tau })d\tau \right).$$ (5)

### Parameter estimation

To estimate the vector of unknown parameters *ω* (this vector can include Cox-regression coefficients *β* and parameters defining the baseline hazard functions *λ*_*i*,*j*_(*t*)) we maximized the log-likelihood function.

The Cox-regression parameters that significantly differed from zero and corresponded to predictive variables were chosen using step-wise regression with backwards elimination. Parameter estimation of the continuous-time Markov chain models with observed covariates in the case of partially observable data have been discussed elsewhere [16], [17]. Since mean times to transition from states 1–8 were relatively small, for parameter estimation we used the model with constant (age-independent) baseline hazard functions and included an additional covariate—the age of patient at arrival in the origin state. Possible time-dependency on the binary (switching) covariates PAD, IHD and CRF have been also taken into account in formulas (4)-(5). Statistical analyses were performed with R version 3.0.1. The estimates of the constant baseline hazard functions refer to the baseline group– 70-year-old-women at arrival in the origin state not suffered from PAD, CHD, and IHD.

## Results

A brief description of the study of patients at first presentation is given in Table 1. Mean patient age at first presentation was 69.0±10.9 years, 59.2% of the patients were male, 56.2% of the patients had a history of previous ulceration, 21.9% of the patients had a minor and 5.0% (n = 13) a major amputation before study inclusion. PAD, CRF and IHD were present at first presentation in 58.1%, 23.5% and 20.0% of the patients, respectively.

There was a higher prevalence of male patients in the studied cohort (*p* = 0.002, exact binomial test) and more than 50% of the male subjects presented with a recurrent diabetic foot ulcer (*p* = 0.03). The mean time of observation was 6.3±5.3 years (range 0.0–16.7). During the observation period, 16.5%, 29.7% and 35.1% of the patients developed PAD, CRF, and IHD, respectively, and 211 patients died (87%).

The number of transitions (or right-censored events on the diagonal) from origin to destination states are given in Table 2.

**Table 2:** Number of transitions. States 1, 3, 5, 7—diabetic foot ulcer. States 2, 4, 6—diabetic foot in remission. State 8 –bilateral major amputation. State 9 –death.

Origin state	Destination state								
	1	2	3	4	5	6	7	8	9
1	0	82	0	20	0	7	0	0	5
2	0	17	294	0	0	0	0	0	64
3	0	293	2	41	0	15	0	2	27
4	0	0	0	19	219	0	0	0	40
5	0	0	0	217	5	15	0	1	28
6	0	0	0	0	0	3	40	0	26
7	0	0	0	0	0	32	1	12	8
8	0	0	0	0	0	0	0	2	13

The maximum likelihood estimates of baseline transition intensities and their confidence intervals for models with/without selection of the covariates are presented in Table 3.

**Table 3:** Estimates of the baseline transition intensities, conditional probabilities of transition, and mean times to transition.

Transition	Approach	Estimates of *λ*_0,*i,j*_	Estimates	Mean time to
		(95% ^1^CI)	of ^2^*ν*_*i,j*_	transition from
				state *i* (years)
1→2	Backward elimination	1.896 (1.401–2.565)	0.810	0.427
	No selection	1.928 (1.296–2.869)	0.889	0.461
1→4	Backward elimination	0.317 (0.205–0.491)	0.135	
	No selection	0.236 (0.090–0.618)	0.109	
1→6	Backward elimination	0.111 (0.053–0.233)	0.048	
	No selection	< 0.001 (0 –Inf)	<0.001	
1→9	Backward elimination	0.016 (0.002–0.153)	0.007	
	No selection	0.004 (0.000–0.044)	0.002	
2→3	Backward elimination	0.307 (0.255–0.371)	0.882	2.871
	No selection	0.289 (0.232–0.360)	0.878	3.040
2→9	Backward elimination	0.041 (0.026–0.064)	0.118	
	No selection	0.040 (0.023–0.068)	0.122	
3→2	Backward elimination	2.478 (2.127–2.887)	0.830	0.335
	No selection	2.757 (2.216–3.431)	0.908	0.329
3→4	Backward elimination	0.313 (0.230–0.425)	0.105	
	No selection	0.222 (0.116–0.425)	0.073	
3→6	Backward elimination	0.089 (0.046–0.173)	0.030	
	No selection	< 0.001 (0 –Inf)	<0.001	
3→8	Backward elimination	0.015 (0.003–0.073)	0.005	
	No selection	< 0.001 (0 –Inf)	<0.001	
3→9	Backward elimination	0.091 (0.046–0.179)	0.031	
	No selection	0.058 (0.023–0.151)	0.019	
4→5	Backward elimination	0.424 (0.352–0.510)	0.895	2.113
	No selection	0.490 (0.368–0.653)	0.950	1.938
4→9	Backward elimination	0.050 (0.030–0.082)	0.105	
	No selection	0.026 (0.011–0.064)	0.050	
5→4	Backward elimination	2.300 (2.007–2.635)	0.924	0.402
	No selection	2.399 (1.828–3.149)	0.953	0.397
5→6	Backward elimination	0.026 (0.004–0.175)	0.010	
	No selection	0.017 (0.001–0.369)	0.007	
5→8	Backward elimination	0.011 (0.001–0.075)	0.005	
	No selection	< 0.001 (0 –Inf)	<0.001	
5→9	Backward elimination	0.151 (0.082–0.279)	0.061	
	No selection	0.100 (0.032–0.315)	0.040	
6→7	Backward elimination	0.370 (0.272–0.505)	0.729	1.967
	No selection	< 0.001 (0 –Inf)	-	-
6→9	Backward elimination	0.138 (0.076–0.249)	0.271	
	No selection	< 0.001 (0 –Inf)	-	
7→6	Backward elimination	1.942 (1.379–2.734)	0.673	0.347
	No selection	2.390 (0.158–36.079)	0.728	0.304
7→8	Backward elimination	0.728 (0.414–1.279)	0.252	
	No selection	0.731 (0.155–3.450)	0.223	
7→9	Backward elimination	0.215 (0.070–0.665)	0.075	
	No selection	0.164 (0.027–1.009)	0.050	
8→9	Backward elimination	0.451 (0.262–0.777)	1.000	2.215
	No selection	0.292 (0.001–165.986)	1.000	3.425

^1^CI–confidence interval

^2^*ν*_*i*,*j*_—conditional probabilities of transition.

As expected, the highest baseline mortality *λ*_0,8,9_ was observed in diabetic foot patients with bilateral major amputation (0.451, CI = (0.262–0.777) in the model with backward elimination and 0.292, CI = (0.001–165.986) in the model without selection of risk factors. Estimates of transition intensities 1→6, 3→6, 3→8, 5→8, 6→7 and 6→9 obtained using an approach without selection of risk factors have abnormally wide confidence intervals. This can indicate that sample size is not large enough or the number of transitions is too small. The mean time to transition from the origin state varied between 0.335 and 2.871 years in the model with backward elimination. The highest probabilities of transition for patients with diabetic foot ulcer corresponded to transitions where there was no change in the level of amputation (none, minor, or major; transitions 1→2, 3→2, 5→4, 7→6).

**Fig 2:**
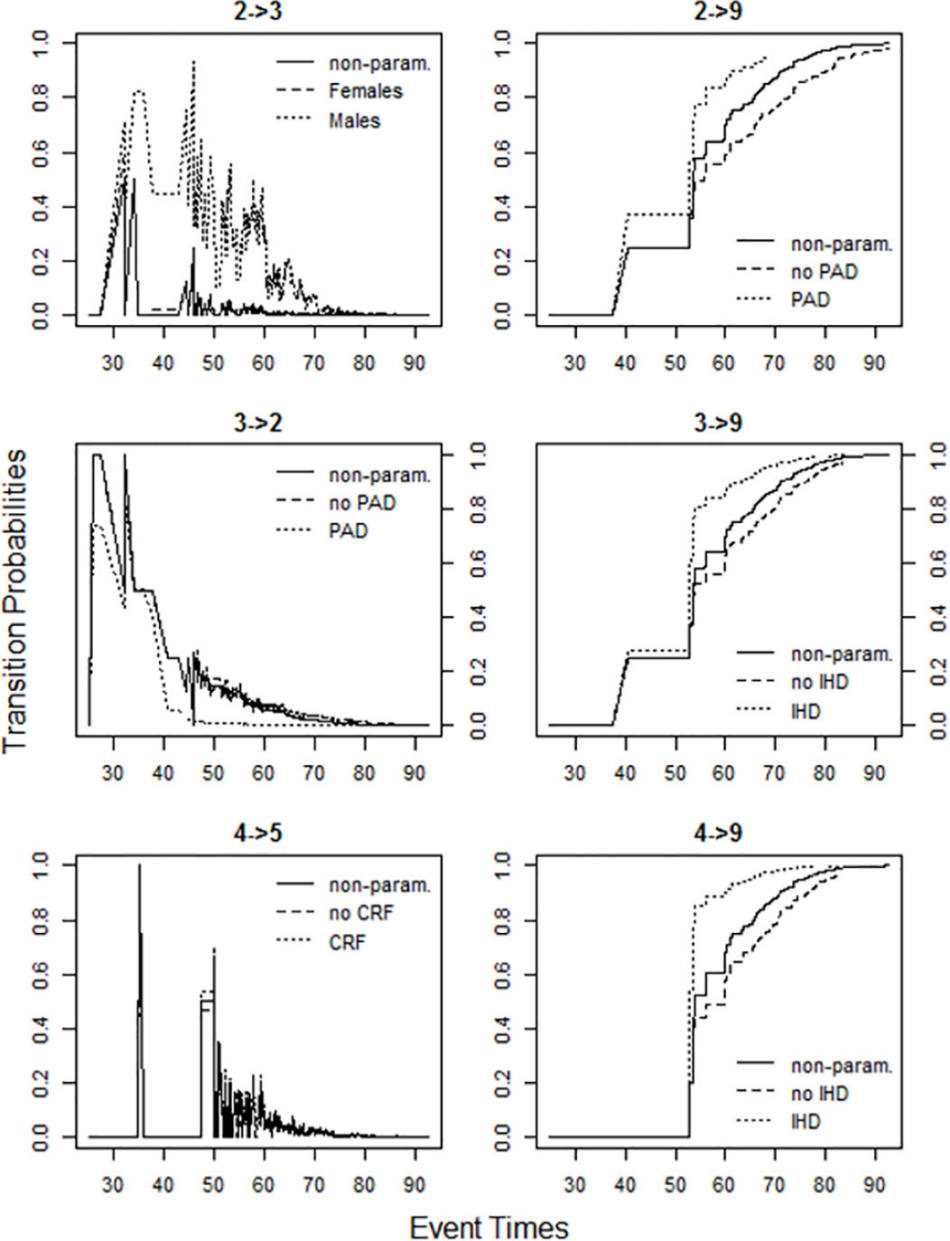
Plot of transition probabilities.

To understand disease progression in diabetes foot patients better, we calculated the marginal (not conditional on any covariates) and adjusted (based on given covariate profile) transition probabilities *P*_*ij*_(0,*t*) from state *i* to state *j* in age interval (0,*t*] subject to independent right censoring and possibly left truncation using the R package mstate [18]. The resulting plot is given in Fig 2.

In each panel of Fig 2 are shown three profiles of transition probabilities–marginal (solid line), for patients from the baseline group (all covariates are equal to zero, dashed line), and for patients with only one risk factor (all but one covariates are equal to zero, dotted line). The first profile refers to the non-parametric model. The second and the third profiles refer to the semi-parametric model. Men with diabetic foot ulcers in remission but without previous amputation(s) (state 2) have a higher risk of developing new ulcers (transition to state 3) compared to women. Patients in remission after previous minor amputation and with chronic renal failure (CRF) have increased risk of new ulceration (transition 4→5). Presence of peripheral arterial disease (PAD) reduces probability of ulcer healing without amputation in patients without any amputation (transition 3→2). PAD and ischaemic heart disease (IHD) increase risk of death in patients in state 2, 3, and 4. These results are in accordance with estimates of the Cox-regression coefficients obtained by using the parametric approach (see Table 4 below).

**Table 4:** Relative risks (RR) of factors and their 95% confidence intervals.

Transition	Approach	Covariates^1^					
		Sex (m/f)	Age (1y)		PAD	CRF	IHD
1→2	No selection	0.99	1.00		0.43*	0.77	1.46
		(0.61–1.63)	(0.98–1.02)		(0.27–0.70)	(0.42–1.43)	(0.85–2.50)
	Backward elimination	-	-		0.49*	-	-
					(0.31–0.76)		
1→4	No selection	0.63	1.00		2.28	2.12	0.43
		(0.25–1.57)	(0.96–1.05)		(0.79–6.61)	(0.75–5.99)	(0.13–1.46)
	Backward elimination	-	-		-	-	-
1→6	No selection	5.03	0.98		4.03·10^7^	5.68	3.10
		(0.09–2.75)	(0.92–1.06)		(0.00-Inf)	(1.17–27.6)	(0.04–2.23)
	Backward elimination		-		-	-	-
1→9	No selection	0.29	1.27*		0.99	1.47	2.24
		(0.04–2.11)	(1.08–1.50)		(0.16–6.01)	(1.90–114.02)	(0.41–12.22)
	Backward elimination	-	1.21*		-	-	-
			(1.04–1.42)				
2→3	No selection	1.38*	1.01*		0.98	1.26	1.05
		(1.08–1.77)	(1.00–1.02)		(0.78–1.24)	(0.96–1.64)	(0.81–1.36)
	Backward elimination	1.39*	1.01*		-	-	-
		(1.09–1.77)	(1.00–1.02)				
2→9	No selection	0.77	1.05*		1.91*	2.38*	1.54
		(0.45–1.33)	(1.02–1.08)		(1.14–3.21)	(1.43–3.95)	(0.90–2.62)
	Backward elimination	-	1.06*		1.76*	2.59*	-
			(1.04–1.09)		(1.06–2.93)	(1.57–4.26)	
3→2	No selection	0.91	1.01		0.72*	0.90	1.16
		(0.71–1.17)	(0.99–1.02)		(0.56–0.92)	(0.69–1.17)	(0.88–1.54)
	Backward elimination	-	-		0.79*	-	-
					(0.63–0.99)		
3→4	No selection	1.05	0.98		1.49	0.85	1.33
		(0.53–2.09)	(0.95–1.00)		(0.76–2.90)	(0.42–1.73)	(0.64–2.79)
	Backward elimination	-	-		-	-	-
3→6	No selection	1.41	1.08*		3.93·10^9^	0.74	0.82
		(0.38–5.18)	(1.01–1.15)		(0.00-Inf)	(0.23–2.38)	(0.25–2.68)
	Backward elimination	-	1.09*		-	-	-
			(1.03–1.16)				
3→8	No selection	0.18	1.10		1.06·10^7^	1.26	3.58·10^7^
		(0.02–1.42)	(0.93–1.29)		(0.00-Inf)	(0.18–8.62)	(0.00-Inf)
	Backward elimination	-	-		-	-	-
3→9	No selection	1.03	1.04*		2.08	2.15*	2.91*
		(0.44–2.42)	(1.00–1.08)		(0.87–4.99)	(1.01–4.57)	(1.29–6.57)
	Backward elimination	-	1.04*		-	2.16*	3.09*
			(1.00–1.09)			(1.002–4.81)	(1.35–7.07)
4→5	No selection	0.88	1.00		0.88	1.39*	1.40*
		(0.65–1.18)	(0.98–1.02)		(0.66–1.17)	(1.05–1.83)	(1.05–1.87)
	Backward elimination	-	-		-	1.38*	1.35*
						(1.05–1.82)	(1.04–1.77)
4→9	No selection	1.30	1.02		1.91	1.18	3.97*
		(0.63–2.67)	(0.98–1.07)		(0.87–4.17)	(0.63–2.20)	(2.08–7.58)
	Backward elimination		-		-	-	4.33*
							(2.29–8.18)
5→4	No selection	0.85	1.00		1.12	0.84	1.18
		(0.63–1.15)	(0.98–1.02)		(0.84–1.49)	(0.64–1.11)	0.89–1.57)
	Backward elimination	-	-		-	-	-
5→6	No selection	3.12	1.03		7.88	0.84	0.57
		(0.65–14.88)	(0.96–1.12)		(0.40–153.82)	(0.27–2.63)	(0.14–2.26)
	Backward elimination	-	-		9.46*	-	-
					(1.36–65.78)		
5→8	No selection	3.07·10^6^	1.02		2.28·10^5^	1.02·10^6^	6.93·10^−11^
		(0.00-Inf)	(0.75–1.39)		(0.00-Inf)	(0.00-Inf)	(0.00-Inf)
	Backward elimination	-	-		-	-	-
5→9	No selection	1.63	1.08*		1.02	1.30	3.14*
		(0.67–3.96)	(1.03–1.14)		(0.43–2.44)	(0.61–2.79)	(1.19–8.32)
	Backward elimination	-	1.08*		-	-	3.53*
			(1.03–1.13)				(1.67–7.46)
6→7	No selection	0.72	1.02		2.64·10^6^	1.38	1.09
		(0.39–1.34)	(0.97–1.07)		(0.00-Inf)	(0.67–2.83)	(0.50–2.38)
	Backward elimination	-	-		-	-	-
6→9	No selection	1.53	1.06*		4.20·10^6^	0.90	3.07*
		(0.65–3.60)	(1.00–1.12)		(0.00-Inf)	(0.35–2.28)	(1.31–7.18)
	Backward elimination	-	1.07*		-	-	2.30*
			(1.02–1.13)				(1.06–5.01)
7→6	No selection	0.87	0.95		1.24	1.15	0.44
		(0.42–1.80)	(0.90–1.01)		(0.09–17.95)	(0.54–2.43)	(0.14–1.43)
	Backward elimination	-	-		-	-	-
7→8	No selection	1.26	0.98		1–01	0.69	1.92
		(0.37–4.28)	(0.89–1.07)		(0.21–4.89)	(0.19–2.54)	(0.46–7.99)
	Backward elimination	-	-		-	-	-
7→9	No selection	0.83	1.14		0.34	2.26	9.13*
		(0.15–4.51)	(0.99–1.31)		(0.06–1.91)	(0.46–11.23)	(2.05–40.77)
	Backward elimination	-	-		-	-	9.12*
							(2.20–37.87)
8→9	No selection	0.61		1.07	0.65	0.99	2.99
		(0.18–2.14)		(0.98–1.17)	(0.00–275.31)	(0.28–3.41)	(0.77–11.58)
	Backward elimination	-		-	-	-	-

^1^All significant factors are marked with a star. Corresponding significance level is equal to 0.05

In Table 4 the relative risks for potential risk factors and their confidence intervals for models with/without selection of the covariates are shown.

Men with diabetic foot ulcers in remission but without previous amputation(s) had a higher risk of developing new ulcers compared to women (RR_23_ = 1.38, CI = (1.09–1.77)). Older age at presentation increased the mortality of patients with diabetic foot ulcers from almost all states (RR_19_ = 1.21, CI = (1.04–1.42), RR_29_ = 1.06, CI = (1.04–1.09), RR_39_ = 1.04, CI = (1.00–1.09), RR_59_ = 1.08, CI = (1.03–1.13), RR_69_ = 1.07, CI = (1.02–1.13)). This factor also increased the probability of unilateral primary major amputation (RR_36_ = 1.09, CI = (1.03–1.16)) and the probability of reulceration in patients in remission without any previous amputation (RR_23_ = 1.01, CI = (1.00–1.02)).

Patients with peripheral arterial disease (PAD) had a 10-fold increased risk of major amputation (RR_56_ = 9.46, CI = (1.36–65.78)), if they had undergone a minor amputation in a previous ulcer episode. Similarly patients with PAD but without any amputation had a significantly reduced probability of ulcer healing without amputation (RR_12_ = 0.49, CI = (0.31–0.76), RR_32_ = 0.79, CI = (0.63–0.99)).

The presence of chronic renal failure increased the mortality of diabetic foot patients without any previous amputation, independent of the absence (RR_29_ = 2.59, CI = (1.57–4.26)), or presence (RR_39_ = 2.16, CI = (1.00–4.81) of active ulceration. This factor also increased the risk of new ulceration in patients in remission after previous minor amputation (RR_45_ = 1.38, CI = (1.05–1.82)). The presence of ischaemic heart disease significantly increased the risk of new ulceration in patients in remission after previous minor amputation (RR_45_ = 1.35, CI = (1.04–1.77) and the risk of death in almost all patients with active ulceration (RR_39_ = 3.09, CI = (1.35–7.07), RR_59_ = 3.53, CI = (1.67–7.46), RR_79_ = 9.12, CI = (2.20–37.87), with the exception of the patients with a first ever foot ulcer.

## Discussion

Although a large proportion of all lower extremity amputations is preceded by diabetic foot ulcers, little is known about the details of progression of the diabetic foot syndrome and the influence of relevant risk factors on its course. Studies on the long-term prognosis of patients with diabetic foot disease show that more than a third of the patients incur a new ulceration within the first 12 months after their index lesion has healed, and 70% experience at least one recurring episode within the next five years [19]. A previous analysis of the cohort described in this paper generated comparable results (1-, 3-, and 5-year cumulative incidences of at least one recurring ulcer episode: 35, 63, and 77%) [20]. Using a multistate Markov chain model to study the course of this chronic condition, we were able to show the high frequency of transitions between active (1, 3, 5 and 7) and inactive stages of disease (2, 4, 6 and 8). We have quantified mean times (in years) to transition for all origin states and conditional probabilities of transition for all possible transitions. We have found that patients with first and with reulceration but without any previous amputation have similar conditional probability of healing without amputation. The later have lower conditional probability of healing with amputation (*ν*_3,4_ + *ν*_3,6_ + *ν*_3,8_ < *ν*_1,4_ + *ν*_1,6_). Conditional probability of death increases with stage in patients with active ulcer (*ν*_1,9_ < *ν*_3,9_ < *ν*_5,9_ < *ν*_7,9_). Conditional probability of death is higher in patients with previous major amputation. Mean time to transition varies between 1.967 and 2.871 years in healed patients and between 0.335 and 0.427 years in patients with active ulcer. The preliminary results obtained in this study are supported by other work previously carried out in this area and are therefore biologically plausible. For example, patients with active ulcers and a previous major amputation had the highest death rates and the presence of other comorbidities had an influence on the probability of healing and survival. Previous studies have not taken into account possibility of developing comorbidities during the observation period. These studies investigated the impact of comorbidities that were present at baseline. With our model we were also able to investigate the impact of comorbidities that were not present at baseline but that developed during the observational period. This enabled us to study the differing impact at various stages of the disease, or more precisely, on the transitions between those stages. For example, ischaemic heart disease (IHD) was a predictor for transition to death from all stages except “diabetic foot ulcer without any previous ulcer” (stage 1), “diabetic foot in remission without prior amputation (stage 2)”, and “bilateral major amputation” (state 8). This observation might explain why some previous studies find an impact of IHD on patients with diabetic foot ulcers while others do not. In our own analysis of this previous cohort [3] and a report from Faglia et al. [20], the presence of IHD was only significant in univariate analysis (HR = 1.59 (1.11–2.28; p<0.05) and HR = 1.892 (1.077–3.324; p = 0.026, respectively), but lost significance in multivariate analysis: On contrast, Izumi et al. reported ischaemic heart disease (IHD) to be the only significant predictor for death in their cohort [21]. The major difference between the studies was, that the latter analyzed first-time amputees with diabetes while the former two followed diabetic foot ulcer cases from all stages (with and without amputation). Other examples are the diverse impacts of age, gender and the presence of peripheral arterial disease (PAD) on specific transitions.

One of the major strengths of our study is the almost complete follow-up over a 17 year long observation period. Among censored patients only 18 patients (6.9%) were unavailable for follow-up before death or the end of the observation period. Furthermore, all data were available with exact dates, enabling us to estimate the time-dependent impact of the potential risk conditions. However, since some transitions occurred rarely (for example transitions 58, 38), we could not obtain the reliable estimates for all Cox-regression coefficients. A further limitation of this study is the fact that all patients in the underlying cohort had experienced at least one active diabetic foot ulcer episode at study inclusion. Therefore, we cannot provide information on transition intensities and relative risk factors for patients with non-ulcerated high risk feet without a previous ulcer episode (“state 0”), which leaves the picture of disease progression in the diabetic foot syndrome somehow incomplete.

To better understand the role of relevant risk factors in disease progression and to increase the power of statistical testing, we need further studies based on a wider range of risk factors and possibly larger data sets. In addition, we plan to validate the model in a population outside that from which it was drawn.

## Conclusions

The diabetic foot is one of the most complex and economically challenging complications of diabetic patients. However, little is known about the dynamics of disease progression and the influence of associated factors. We proposed a nine-state continuous–time Markov chain model for quantifying disease progression and the influence of time-dependent factors. We applied our model to the long-term data of 260 patients with diabetic foot ulcer collected in a single specialized center in Germany. Our study combines two important properties that have not been observed in previous investigations of the diabetic foot patients–the long observation period (17-year follow up) and a detailed subdivision of the patients in groups with respect to their amputation (none, minor or major) and health (ulceration or remission) status. It has allowed us to carry out a more detailed analysis of the disease progression and the influence of relevant risk factors in these patients. We have investigated the differing impact of comorbidities (PAD, IHD, CRF) at various stages of diabetic foot. The model is therefore helpful to understand the complex interplay of active and inactive episodes of diabetic foot disease and will now be used to comprehensively analyze larger prospective datasets from patients with diabetes and lower extremity complications.
